# Long-term Risk of Epilepsy Following Invasive Group B *Streptococcus* Disease in Neonates in Denmark

**DOI:** 10.1001/jamanetworkopen.2023.9507

**Published:** 2023-04-21

**Authors:** Malene Risager Lykke, Henrik Toft Sørensen, Joy Elisabeth Lawn, Erzsébet Horváth-Puhó

**Affiliations:** 1 Department of Clinical Epidemiology, Aarhus University and Aarhus University Hospital, Aarhus, Denmark; 2 Faculty of Epidemiology and Population Health, London School of Hygiene & Tropical Medicine, London, United Kingdom; 3 Maternal, Adolescent, Reproductive & Child Health (MARCH) Centre, London School of Hygiene & Tropical Medicine, London, United Kingdom

## Abstract

**Importance:**

The risk of epilepsy after neonatal invasive Group B *Streptococcus* (iGBS) disease, particularly iGBS sepsis, is poorly understood.

**Objective:**

To examine the association between neonatal iGBS (sepsis or meningitis) and long-term risk of epilepsy, stratified by sex, prematurity, and maternal socioeconomic position (SEP).

**Design, Setting, and Participants:**

This population-based cohort study was conducted in Denmark with an inclusion period from 1997 through 2017 and follow-up until the end of 2018. A general population comparison cohort was randomly sampled and matched up to 10:1 to the exposed cohort. Linkage between Danish national registers were applied for data collection. Participants were infants aged 0 to 89 days. The general population comparison cohort was matched by sex, the child’s year and month of birth, and gestational age. SEP was defined by maternal income and education.

**Exposure:**

Hospital-diagnosed iGBS (sepsis or meningitis) during the first 89 days after birth.

**Outcomes and measures:**

Epilepsy was defined by *International Statistical Classification of Diseases and Related Health Problems, Tenth Revision* codes and/or prescription codes for antiepileptic drugs using Danish medical registry data. Cumulative risk (CR) of epilepsy was calculated by treating death as a competing event. Cox proportional hazards regression was used to estimate hazard ratios with 95% CIs. Effect modification by sex, prematurity, and maternal SEP was assessed on an additive scale.

**Results:**

A total of 1432 children (792 [55.3%] boys; 1126 [78.6%] with gestational age ≥37 weeks) were identified with iGBS disease: 1264 with sepsis and 168 with meningitis. In the comparison cohort, there were 14 211 children (7869 [55.4%] boys; 11 260 [79.2%] with gestational age ≥37 weeks). The overall (0 to 22 years) CR of epilepsy was 3.6% (95% CI, 2.6%-5.0%) in children with iGBS disease and 2.3% (95% CI, 1.9%-2.7%) in the comparison cohort. The overall CR of epilepsy for iGBS meningitis was 15.1% (95% CI, 8.9%-22.8%) and 2.2% (95% CI, 1.4%-3.4%) for iGBS sepsis. The adjusted hazard ratio for epilepsy in children with iGBS disease was 2.04 (95% CI, 1.46-2.85). Being a boy, born premature, or born to a mother belonging to a low SEP group was associated with an increased risk of epilepsy in later childhood.

**Conclusion:**

In this population-based cohort study of 1432 neonates, iGBS disease was associated with a higher incidence of epilepsy in later childhood, notably after meningitis. Premature birth, sex, and low maternal SEP modified the association.

## Key Points

**Question:**

Is neonatal invasive Group B *Streptococcus* (iGBS) sepsis or meningitis associated with increased risk of epilepsy?

**Findings:**

This cohort study of children born in Denmark from 1997 through 2017 found children diagnosed with iGBS meningitis were associated with a significantly higher risk of epilepsy than the matched comparison cohort. The cumulative risk of epilepsy for iGBS sepsis resembled that of the comparison cohort.

**Meaning:**

These findings suggest that epilepsy should be added to the list of neurological outcomes following iGBS disease in early infancy, especially meningitis.

## Teaser

This cohort study investigates the association between neonatal invasive group B *Streptococcus* and long-term risk of epilepsy, stratified by sex, prematurity, and maternal socioeconomic position.

## Introduction

Invasive Group B *Streptococcus* (iGBS) disease remains a leading cause of neonatal and young infant mortality worldwide.^1,2,3,4,5,6^ Approximately 20 million pregnant women were estimated to have rectovaginal colonization with Group B *Streptococcus* (GBS) in 2020.^7^ Globally in 2020, an estimated 500 000 cases of iGBS disease occurred within the first 3 months after birth.^7^ iGBS disease has been associated with maternal death, stillbirth, neonatal death, and neurodevelopmental impairment (NDI) in iGBS survivors,^7,8,9^ including neonatal stroke, encephalopathy, cerebral palsy, intellectual and/or motor, vision, or hearing impairment.^1,2,8,9,10,11,12,13,14,15,16,17,18,19^ Studies on NDI outcomes after iGBS disease have often been small and mainly focused on meningitis.^9,14^ This is evident from a systematic review and meta-analyses including 18 studies on neonatal iGBS and NDI outcomes. Studies about iGBS sepsis were not included because they were too few.^14^ The study populations included 7 to 103 patients and a median follow-up time from 0.75 to 10.5 years, of which 11 studies had a median follow-up time of less than 5 years.^14,20^ Studies with larger numbers of exposed children and longer follow-up time will be needed to further evaluate neurological outcomes after iGBS. Epilepsy has been reported as an outcome after iGBS disease, notably meningitis, but the risk of epilepsy after neonatal iGBS disease is still to be investigated further.^16,19,21^

Thus, a knowledge gap remains regarding iGBS disease and the risk of epilepsy, particularly the association between iGBS sepsis and the long-term risk of epilepsy. Therefore, we investigated the risk of epilepsy in infants diagnosed with iGBS sepsis or meningitis during the first 3 months of age, and we examined whether the child’s sex, prematurity, and the mother’s socioeconomic position (SEP) modified the risk of epilepsy after iGBS disease.

## Methods

### Data Sources, Design, and Study Cohorts

We conducted this nationwide, matched cohort study including all children born in Denmark from January 1, 1997, to December 31, 2017,^22^ with follow-up until December 31, 2018. Using the unique personal identification number, we were able to link data from various health registries in Denmark (eAppendix 2 in Supplement 1).

### Ethics and Informed Consent From Study Participants

All data were analyzed at Statistics Denmark by using encrypted identification numbers; no contact was made with individuals. By Danish law, analyses of anonymous data do not require ethical review board approval nor informed consent from participants. The study was approved by the Danish Data Protection Agency. This study followed the Strengthening the Reporting of Observational Studies in Epidemiology (STROBE) and the Reporting of Studies Conducted Using Observational Routinely-Collected Data (RECORD) reporting guidelines for cohort studies.

### Invasive Group B *Streptococcus* Cohort

Exposed children were defined as those diagnosed with iGBS sepsis or meningitis within the first 89 days of age (*International Statistical Classification of Diseases and Related Health Problems, Tenth Revision [ICD-10]* codes are listed in eAppendix 1 in Supplement 1; screening strategy is described in eAppendix 5 in Supplement 1). The index date was defined as the date of hospital admission for iGBS.

### General Population Comparison Cohort

The comparison cohort was randomly selected and matched to each exposed infant at a ratio of up to 10:1 by sex, year and month of birth, and gestational age (<28 weeks, 28-36 weeks, and ≥37 weeks). Children in the comparison cohort had no history of iGBS disease in the first 89 days of life. The index date for the comparison cohort was the same as the corresponding admission date of the child with iGBS disease.

### Registry Linkage

The cohorts were established by linking the Danish Medical Birth Registry (DMBR),^23,24^ the Danish Civil Registration System (CRS),^25^ the Danish National Patient Registry (DNPR),^26^ and the Psychiatric Central Research Register (PCRR).^27,28^ The Danish National Prescription Registry (NPR) records data on prescription drugs. The *ICD-10* hospital codes for mental, behavioral, and nervous system disorders were obtained from the DNPR and the PCRR and were used together with prescriptions data (Anatomical Therapeutic Chemical Classification System codes) to identify children as well as mothers with epilepsy.^26,29^ Data on maternal income and educational level were collected from the Integrated Database for Labor Market Research and the Educational Attainment Register^30^ (overview of registries, variables, and usage is found in eAppendix 2 in Supplement 1). Education was divided into low (primary or lower education), middle (upper secondary or academic professional degree), and high (university education at bachelor’s degree or higher).

### Epilepsy

We assessed epilepsy as an overall outcome defined by *ICD-10* codes and/or prescription codes for antiepileptic drugs (eAppendix 1, eTable 1, and eTable 2 in Supplement 1). The 3 types of epileptic seizures (generalized, focal, and unknown onset) were examined as separate outcomes in a sensitivity analysis. The diagnostic criteria for epilepsy in Denmark followed the International League Against Epilepsy and the International Bureau for Epilepsy’s guidelines and consensus definitions for epileptic seizure and epilepsy.^31,32,33^

### Covariates

Information regarding maternal characteristics, including age, delivery type, and history of epilepsy, was analyzed. These data were obtained from the CRS, the DNPR, and the DMBR as well as the Integrated Database for Labour Market Research and the Educational Attainment Register.^30^

### Statistical Analyses

Both cohorts were followed up from the index date until epilepsy diagnosis or redemption of a prescription for an antiepileptic drug, date of death, emigration, or the end of the study period (December 31, 2018), whichever occurred first. The maximum follow-up time was 22 years. We calculated the overall cumulative risk (CR) of epilepsy during 22 years of follow-up, treating death as a competing event. Incidence rates (IRs) per 1000 person-years (PY) were computed in the iGBS cohort and the comparison cohort. Cox proportional hazards regression analysis was used to compute hazard ratios (HRs) including 95% CIs. Statistically significant results were considered as *P* < .05 and 95% CI. HRs were computed for the overall follow-up period (0 to 22 years) and stratified by follow-up time (0 to 5 years and >5 to 22 years). HRs were adjusted for sex, prematurity, year of birth, and maternal income categories. Missing incomes were included in a separate income category. We used directed acyclic graphs to identify relevant covariates for adjustment (see eAppendix 3 in Supplement 1). A sensitivity analysis was performed using a case definition of epilepsy solely based on *ICD-10* codes obtained from the DNPR to test the robustness of our outcome to potential misclassification. We conducted stratified analyses according to the child’s sex, premature birth (<37 weeks of gestational age), maternal age, and maternal SEP in terms of income and educational level.

We evaluated the extent to which the child’s sex and gestational age and the mother’s SEP modified the association of iGBS disease with the risk of epilepsy.^34,35^ In our calculations, a girl with no iGBS disease, a child born at term with no iGBS disease, and a low level of maternal education were used as reference groups. We assessed potential effect measure modification on an additive scale to identify subgroups estimating a higher risk of epilepsy after iGBS disease during infancy (eAppendix 4 in Supplement 1). Effect modification on an additive scale occurs when differences in rates between the exposed and unexposed groups (herein, children with and children without a history of iGBS disease, respectively) differ by strata of a third variable (hence, the effect modifier: sex, prematurity, or SEP). We calculated effect modification as an interaction contrast (IC) assessing the extent to which sex, prematurity, or SEP occurring simultaneously with iGBS disease was a predisposing factor for epilepsy. The IC disaggregates the rate of epilepsy into the following components: (1) the rate changes associated with iGBS disease only, (2) the rate changes associated with an effect modifier, and (3) the general population rate among children with no iGBS disease and no effect modifier. If the rate among the doubly exposed patients (those with both iGBS disease and an effect modifier) is equal to the sum of the components (IC = 0), there is no evidence of a biological interaction between iGBS disease and the potential effect modifier. If the rate among doubly exposed patients is greater than the sum of the components (IC >1), iGBS disease and the effect modifier are associated with a synergistically increased rate of epilepsy beyond the sum of their individual effects. For example, the IC for prematurity was calculated as IC_Prematurity_ = IR_iGBS/Premature_ – IR_iGBS/Term_ – IR_Non-iGBS/Premature_ + IR_Non-iGBS/Term_. In addition, we calculated the attributable proportion (AP), which resembles the proportion of the combined effect associated with the interaction (ie, for prematurity: AP_Prematurity_ = IC_Prematurity_/IR _iGBS/Premature_. ICs were calculated on the basis of IRs per 1000 PY in the first 5 years of follow-up and in the total follow-up period (0 to 22 years). Low SEP was defined as low level of maternal education in this analysis.

Statistical analyses were performed from April through October 2022. SAS version 9.4 (SAS Institute) was used for statistical analyses.

## Results

We identified 1432 children (792 [55.3%] boys; 1126 [78.6%] with gestational age ≥37 weeks) with a history of iGBS disease (1264 [88.3%] with sepsis and 168 [11.7%] meningitis) and 14 211 children (7869 [55.4%] boys; 11 260 [79.2%] with gestational age ≥37 weeks) with no history of iGBS disease from a source population consisting of 1 297 383 liveborn children in the period from 1997 through 2017 in Denmark (Table 1). The iGBS cohort was followed for a median (IQR) of 12.6 (6.3-18.0) years, and the comparison cohort members were followed for a median (IQR) of 13.3 (7.1-18.2) years.

**Table 1.** Characteristics of Children With iGBS Disease and a Matched Comparison Cohort

Characteristic	Children, No. (%)	
	iGBS cohort (n = 1432)	Comparison cohort (n = 14 211)
iGBS type		
Meningitis	168 (11.7)	NA
Sepsis	1264 (88.3)	NA
Sex		
Female	640 (44.7)	6342 (44.6)
Male	792 (55.3)	7869 (55.4)
Gestational age, wk		
<28	50 (3.5)	391 (2.8)
28-36	256 (17.9)	2560 (18)
≥37	1126 (78.6)	11 260 (79.2)
Year of iGBS		
1997-2001	478 (33.4)	4748 (33.4)
2002-2006	326 (22.8)	3241 (22.8)
2007-2011	326 (22.8)	3231 (22.7)
2012-2017	302 (21.1)	2991 (21.0)
Multiplicity		
Singleton	1334 (93.2)	13 031 (91.7)
Twins	90 (6.3)	1138 (8.0)
Higher order	8 (0.6)	42 (0.3)
Birth weight, g		
<2500	243 (17.0)	2013 (14.2)
2500-2999	152 (10.6)	1951 (13.7)
3000-3499	330 (23.0)	3999 (28.1)
3500-3999	411 (28.7)	4008 (28.2)
4000-4499	245 (17.1)	1822 (12.8)
≥4500	51 (3.)	418 (2.9)
Maternal age, y		
18-24	197 (13.8)	1863 (13.1)
25-29	468 (32.7)	4770 (33.6)
30-34	505 (35.3)	4966 (34.9)
35-39	221 (15.4)	2187 (15.4)
≥40	41 (2.9)	425 (3.0)
Parity		
1	795 (55.5)	6526 (45.9)
≥2	623 (43.5)	7526 (53.0)
Missing	14 (1.0)	159 (1.1)
Delivery type		
Vaginal	1016 (70.9)	10 900 (76.7)
Cesarean	416 (29.1)	3311 (23.3)
Maternal epilepsy before index date		
Yes	29 (2.0)	166 (1.2)
No	1403 (98)	14 045 (98.8)
Maternal income		
Low	375 (26.2)	3521 (24.8)
Middle	361 (25.2)	3524 (24.8)
Medium high	347 (24.2)	3522 (24.8)
High	334 (23.3)	3550 (25.0)
Missing	15 (1.0)	94 (0.7)
Maternal education		
Low (primary or lower education)	444 (31.0)	4548 (32.0)
Middle (upper secondary or academic professional degree)	716 (50.0)	7072 (49.8)
High (university education at bachelor degree or higher)	168 (11.7)	1714 (12.1)
Missing	104 (7.3)	877 (6.2)

Abbreviations: iGBS, invasive group B *Streptococcus*; NA, not applicable.

### CR of Epilepsy in Children With iGBS Disease and in Comparison Cohort

**Figure 1.**
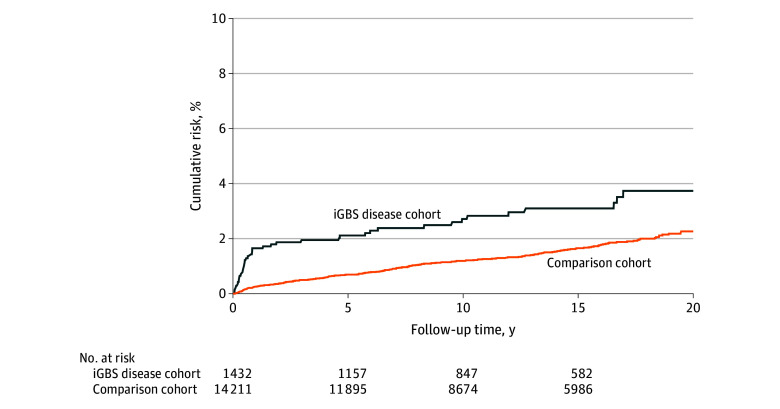
Cumulative Risk of Epilepsy According to Follow-up Time in Years Numbers at risk at index date and after 5, 10, and 15 years of follow-up for the exposed (with invasive group B *Streptococcus *[iGBS]) and comparison cohort (without iGBS).

The overall CR of epilepsy in children with iGBS disease was 3.6% (95% CI, 2.6%-5.0%), compared with 2.3% (95% CI, 1.9%-2.7%) in the comparison cohort (Figure 1). Children with iGBS meningitis had an overall CR of 15.1% (95% CI, 8.9%-22.8%), compared with 1.4% (95% CI, 0.8%-2.3%) in the comparison cohort. Children with iGBS sepsis had a CR of 2.2% (95% CI, 1.4%-3.4%) compared with 2.4% (95% CI, 2.0%-2.8%) in the comparison cohort.

### IR and HR 

The overall IR for epilepsy in children with iGBS disease was 2.4 per 1000 PY (95% CI, 1.7-3.1 per 1000 PY), compared with 1.2 per 1000 PY (95% CI, 1.0-1.3 per 1000 PY) in the comparison cohort (Table 2). The overall HR adjusted for sex, prematurity, year of birth, and maternal income was 2.04 (95% CI, 1.46-2.85) (Table 2).

**Table 2.** Rate of Epilepsy Among Children With iGBS and in the Comparison Cohort, Denmark, 1997 to 2017

Overall outcome in given period	iGBS cohort			Comparison cohort			Adjusted HR (95% CI)^a^
	No. of outcome events	Sum of person-years	IR per 1000 person-years (95% CI)	No. of outcome events	Sum of person-years	IR per 1000 person-years (95% CI)	
iGBS total							
0-22 y	41	17 180	2.39 (1.66-3.12)	204	175 460	1.16 (1.00-1.32)	2.04 (1.46-2.85)
0-5 y	29	6420	4.52 (2.87-6.16)	92	65 370	1.41 (1.12-1.69)	3.18 (2.09-4.82)
>5-22 y	12	10 760	1.12 (0.48-1.75)	112	110 090	1.02 (0.83-1.21)	1.09 (0.60-1.98)
iGBS sepsis							
0-22 y	20	15 640	1.28 (0.72-1.84)	189	157 010	1.20 (1.03-1.38)	1.06 (0.67-1.67)
iGBS meningitis							
0-22 y	21	1540	13.62 (7.79-19.44)	15	18 450	0.81 (0.40-1.22)	15.68 (8.07-30.49)

Abbreviations: HR, adjusted hazard ratio; iGBS, invasive group B *Streptococcus*; IR, incidence rate.

^a^ HR adjusted for sex, prematurity, year of birth, and maternal income.

The 5-year IR for epilepsy in children with iGBS disease was 4.5 per 1000 PY (95% CI, 2.9-6.2 per 1000 PY), then declined to 1.1 per 1000 PY (95% CI, 0.5-1.8 per 1000 PY) in the remaining follow-up period. The comparison cohort had an IR of 1.4 per 1000 PY (95% CI, 1.1-1.7 per 1000 PY) in the first 5 years of life and an IR of 1.0 per 1000 PY (95% CI, 0.8-1.2) in the remaining follow-up period. The adjusted HR for epilepsy during the first 5 years of life was 3.18 (95% CI, 2.10-4.83).

For children with iGBS meningitis, the overall IR was 13.6 per 1000 PY (95% CI, 7.8-19.4 per 1000 PY) and the adjusted HR was 15.68 (95% CI, 8.07-30.49). For children with iGBS sepsis, the overall IR was 1.3 (95% CI, 0.7-1.8), and the adjusted HR was 1.06 (95% CI, 0.67-1.67) (Table 2).

### Sensitivity Analyses

When epilepsy was defined solely on the basis of *ICD-10* codes, the overall CR among children with iGBS disease was 3.2% (95% CI, 2.2%-4.4%), the IR was 2.1 per 1000 PY (95% CI, 1.4-2.8 per 1000 PY), and the adjusted HR was 2.15 (95% CI, 1.50-3.08) (eTable 3 in Supplement 1). For children with iGBS disease, no appreciable differences in risk were observed among the 3 types of epilepsy: the HR for general onset was 2.81 (95% CI, 1.34-5.87), the HR for focal onset was 2.67 (95% CI, 1.16-6.13), and the HR for unknown onset was 1.83 (95% CI, 1.14-2.94).

### Stratified Analyses

**Figure 2.**
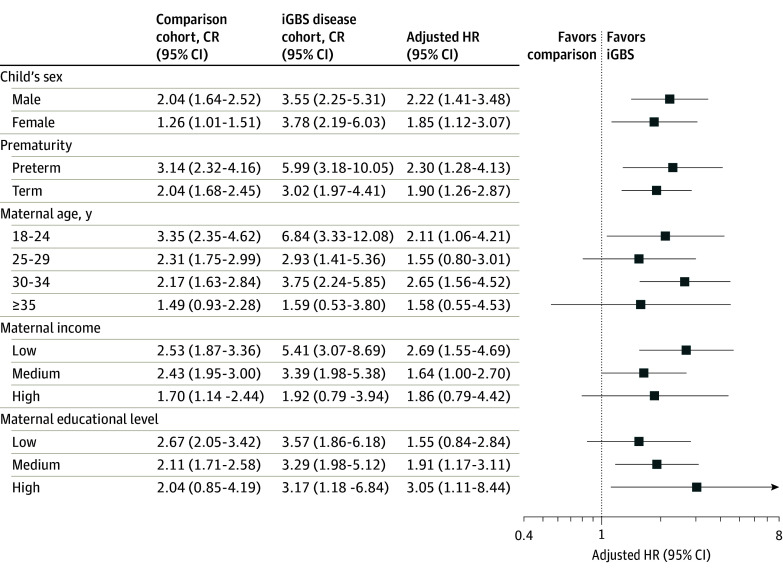
Analyses Stratified by Children’s Characteristics and Maternal Socioeconomic Position The figure shows cumulative risk (CR) with 95% CIs for the iGBS and comparison (non-iGBS) cohorts and the adjusted HRs with 95% CIs. HR indicates the adjusted hazard ratio, where we have adjusted for sex, prematurity, year of birth, and maternal income with 95% CI; iGBS, invasive group B *Streptococcus*.

Stratified by sex, boys had an overall CR of 3.5% (95% CI, 2.3%-5.3%), whereas girls had a CR of 3.8% (95% CI, 2.2%-6.0%) (Figure 2). The overall IRs were similar for both sexes: IR for boys was 2.4 per 1000 PY (95% CI, 1.4-3.4 per 1000 PY) and for girls was 2.4 per 1000 PY (95% CI, 1.3-3.5 per 1000 PY). The adjusted HR for boys was 2.22 (95% CI, 1.41-3.48), whereas the adjusted HR for girls was 1.85 (95% CI, 1.12-3.07).

Stratified by gestational age, children born premature with iGBS disease had a CR of 6.0% (95% CI, 3.2%-10.0%), compared with 3.1% (95% CI, 2.3%-4.2%) for the children born prematurely from the general population comparison cohort. The overall IR was higher in the children born prematurely, namely 4.0 per 1000 PY (95% CI, 1.9-6.2 per 1000 PY), compared with 1.7 per 1000 PY (95% CI, 1.2-2.1 per 1000 PY) in the children born prematurely from the comparison cohort. The adjusted HR among children born prematurely was 2.30 (95% CI, 1.28-4.13), compared with an adjusted HR of 1.90 (95% CI, 1.26-2.87) for children born at term (Figure 2).

Stratified by SEP (measured by maternal income), overall CR and IR of epilepsy were higher than those in the comparison cohort if the mother was categorized in the low-income group (Figure 2). The overall IR of epilepsy in the iGBS cohort was 3.6 per 1000 PY (95% CI, 1.8-5.3 per 1000 PY) for children whose mother was in the low-income group, compared with 1.5 per 1000 PY (95% CI, 0.3-2.7 per 1000 PY) for those with a mother in the high-income group. The adjusted HR of epilepsy was 2.69 (95% CI, 1.55-4.69) in the low-income group and 1.86 (95% CI, 0.79-4.42) in the high-income group. The low-education group had an IR of 2.0 per 1000 PY (95% CI, 0.9-3.2 per 1000 PY) in the iGBS cohort (adjusted HR, 1.55 [95% CI, 0.84-2.84]), whereas the high-education group had an IR of 3.0 per 1000 PY (95% CI, 0.4-5.6 per 1000 PY) and an adjusted HR of 3.05 (95% CI, 1.11-8.44) (Figure 2).

### Effect Modification Analyses

**Figure 3.**
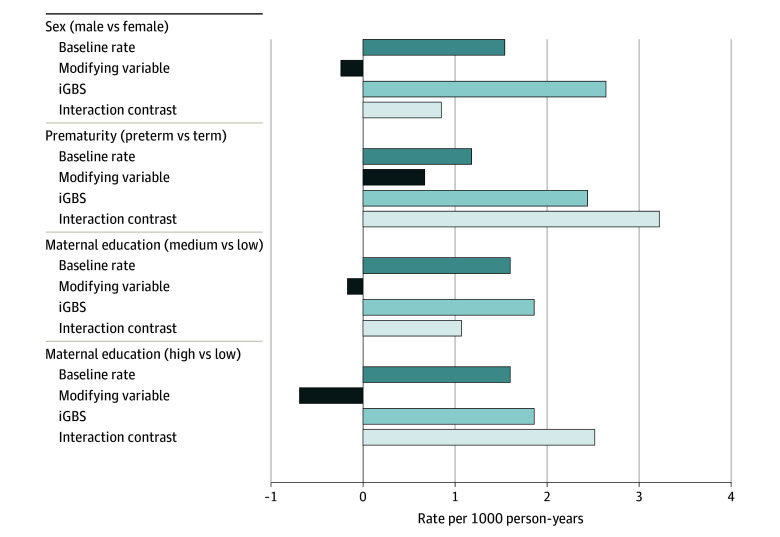
Effect Modification Analysis Proportion of the incidence rate per 1000 person-years attributable to baseline rate, modifying variable alone (sex, prematurity, maternal education), invasive group B *Streptococcus* (iGBS) disease alone, and the interaction.

During the first 5 years of follow-up, a positive IC was found in boys vs girls (IC, 0.85 [95% CI, −2.48 to 4.19]; AP, 15.9%), prematurity (preterm vs term: IC, 3.22 [95% CI −1.85 to 8.29]; AP, 42.4%), and low maternal level of education (medium vs low: IC, 1.07 [95% CI, −2.43 to 4.56]; AP, 26.5%; and high vs low: IC, 2.52 [95% CI, −3.33 to 8.37]; AP, 47.7%) (Figure 3; eTable 4 in Supplement 1). For the overall follow-up period, the IC was also positive (eTable 5 in Supplement 1).

## Discussion

In this large, matched cohort study from Denmark on children with neonatal iGBS sepsis or meningitis, we found a significantly higher risk of epilepsy in children with iGBS meningitis in later childhood compared with children not exposed to iGBS. The risk of epilepsy for iGBS sepsis was not significant. The cumulative incidence of epilepsy was higher in the first 5 years after diagnosis of iGBS compared with the children not exposed to iGBS. Our data provide evidence of an effect modification associated with epilepsy risk after iGBS disease by sex, gestational age, and low maternal SEP, on an additive scale. To our knowledge, this is the first study to examine the long-term risk of epilepsy as an individual outcome after iGBS sepsis or meningitis.

### Comparison With Other Studies

To our knowledge, seizures or epilepsy have been reported as outcomes after iGBS disease in 3 smaller studies.^16,19,21^ A recent observational study from the UK including 119 infants described seizures as an outcome 2 to 5 years following iGBS sepsis or meningitis. Four children had seizure as outcome, 1 after sepsis and 3 after meningitis.^21^ A retrospective cohort study from Taiwan included 145 youths aged 0 to 18 years with previous brain infections due to either bacterial or viral agents. Of these, 28 had iGBS disease, but age of onset was not further specified. Epilepsy was assessed for brain infections and 2 of the 28 youths with iGBS were diagnosed with epilepsy in the follow-up period.^19^ An Australian study investigated death and hospitalization in infants affected with neonatal iGBS. They found 1.5% (57 of 3721 patients) of iGBS readmissions to be associated with epilepsy.^16^ The age at readmission and the exposure (iGBS sepsis, meningitis, or pneumonia) of these children were not further clarified. In combination with the review and meta-analysis of NDI outcomes after iGBS disease,^14^ the knowledge gaps concerning iGBS research concern study size and length of follow-up as well as the assessment of iGBS sepsis cases. In our study, the study size and length of follow-up exceed previous studies looking at seizures and/or epilepsy as outcome measures.

The main strength of this study is the use of routinely collected registry data of high quality and the complete long-term follow-up. Our study addresses an important epidemiological question, particularly for iGBS sepsis, which only a few studies have examined. We were able to include a large cohort of children with iGBS disease and a large comparison cohort of children without a history of iGBS disease and conduct long-term follow-up until adolescence. Our study design with a matched comparison cohort was successful, because we were able to match on all matching variables with small variances between the iGBS cohort and comparison cohort, which reduced any residual confounding. Another strength is the assessment of attributable risk factors for epilepsy with the calculation of interaction contrast and estimation of effects in different strata (eg, sex or preterm birth). Furthermore, our sensitivity analysis underlined our findings when the outcome was assessed using *ICD-10* codes only.

The epilepsy diagnosis codes have previously been validated in the DNPR and the PCRR with a positive predictive value of 81% (95% CI, 75%-87%).^36^ Furthermore, the CR of our comparison cohort members resembled that of the study on incidence and prevalence of epilepsy in Denmark.^37^ We cannot exclude bias due to missing data (eg, on education and income or correct gestational age), but they are not likely to have much of an effect on our results.

### Limitations

Our study also has limitations. We might potentially have included some children with iGBS disease not diagnosed by culture, or we might have missed children with iGBS disease who were not given the correct diagnostic code. However, because such misclassification would probably have been nondifferential, it would have led to bias toward the null. Furthermore, the date of iGBS onset was defined based on the admission date, which means that we might have missed some iGBS infections from the day of birth. Our cohort of iGBS meningitis was small, so even though the risk was statistically significantly increased, our estimates must be interpreted with caution. We chose to adjust for prematurity although we cannot know whether iGBS was a consequence of or a risk factor for premature birth in this study.

We did not account for childhood accidents (including maternal or childhood drug use), infectious diseases (including meningitis of any pathogen), or head trauma after index date. Our comparison cohort might have consisted of children with neonatal stroke or cerebral palsy, leading to epilepsy. Because the differences in cumulative risk is most marked in the first 2 years of life, we do not expect the exposures not accounted for to contribute substantially to our findings.

Unmeasured confounding factors may have created bias away from the null. Such factors might have included racial differences because the susceptibility to GBS carriage has been found to be greater in some regions (eg, Southeast Asia) than Europe.

SEP consists of several elements, which can be difficult to define using a few variables. Because the mothers of the children in our cohorts represented a young adult population, level of education was found to be a good measure for SEP as it is obtained earlier in life; however, use of this variable might have introduced some misclassification. Other categorizations might possibly have shed light on other aspects of SEP and although the lower bound of our 95% CI had the same direction, a larger sample size would have increased the precision of our estimates.

Identifying possible subgroups of people who would benefit the most from an intervention is always of importance. Our results show an increased risk of epilepsy among boys with iGBS disease, which could inform programmatic targeting and raise further research questions on the mechanism of these sex differences.

## Conclusion

This cohort study found that iGBS disease, particularly meningitis, was associated with an increased risk of epilepsy in later childhood. Premature birth, sex, and low maternal SEP were found to be effect modifiers. Our findings have implications for estimating the global burden of iGBS and should be considered in relation to the cost-effectiveness of interventions, such as intrapartum antibiotic prophylaxis and maternal vaccination. Importantly, these data also have implications for affected individuals and underline the need for better long-term follow-up and care.

## Notes

Supplement 1. **eAppendix 1.** Diagnostic Code Numbers of Group B *Streptococcus* and Anatomical Therapeutic Chemical Classification System Codes of Group B *Streptococcus* Illnesses, Epilepsy, and Anti-Epileptic Drugs **eTable 1.** Overview of Exposure and Outcome-Codes **eTable 2.** Overview of Antiepileptic Drugs Included in This Study, ATC Code and Names (Clobazam Not Included) **eAppendix 2.** Data Sources **eAppendix 3.** Directed Acyclic Graph (DAG) **eAppendix 4.** Measures of Interaction on an Additive Scale **eAppendix 5.** Screening and Treatment for Suspected GBS Infection During Pregnancy or After Birth **eTable 3.** Incidence Rates of Epilepsy Among Children With iGBS and in the Comparison Cohort, With Epilepsy Defined Based on ICD-10 Codes Only **eTable 4.** Effect Modification by Sex and Gestational Age of the Association Between Invasive Group B *Streptococcus* Disease and Epilepsy 0–5 Years After Birth **eTable 5.** Effect Modification by Sex, Gestational Age, and Maternal Level of Education of the Association Between Invasive Group B *Streptococcus* Disease and Epilepsy 0–22 Years After Birth
eReferences

Supplement 2.
Data Sharing Statement
