# Error in Figure 2

**DOI:** 10.1001/jamanetworkopen.2024.42173

**Published:** 2024-10-04

In the Original Investigation titled “Long-term Risk of Epilepsy Following Invasive Group B *Streptococcus* Disease in Neonates in Denmark,”^1^ published April 21, 2023, there were 2 column headers incorrectly swapped in Figure 2: “iGBS disease cohort, CR (95% CI)” and “Comparison cohort, CR (95% CI).” This article has been corrected.^1^
