# Supplementary material for: Long-term Risk of Epilepsy Following Invasive Group B Streptococcus Disease in Neonates in Denmark
Source: JAMA Netw Open. 2023 Apr 21;6(4):e239507. doi: 10.1001/jamanetworkopen.2023.9507 (PMC10122176; doi:10.1001/jamanetworkopen.2023.9507)
Supplement: Supplement 1. — eAppendix 1. Diagnostic Code Numbers of Group B Streptococcus and Anatomical Therapeutic Chemical Classification System Codes of Group B Streptococcus Illnesses, Epilepsy, and Anti-Epileptic Drugs eTable 1. Overview of Exposure and Outcome-Codes eTable 2. Overview of Antiepileptic Drugs Included in This Study, ATC Code and Names (Clobazam Not Included) eAppendix 2. Data Sources eAppendix 3. Directed Acyclic Graph (DAG) eAppendix 4. Measures of Interaction on an Additive Scale eAppendix 5. Screening and Treatment for Suspected GBS Infection During Pregnancy or After Birth eTable 3. Incidence Rates of Epilepsy Among Children With iGBS and in the Comparison Cohort, With Epilepsy Defined Based on ICD-10 Codes Only eTable 4. Effect Modification by Sex and Gestational Age of the Association Between Invasive Group B Streptococcus Disease and Epilepsy 0–5 Years After Birth eTable 5. Effect Modification by Sex, Gestational Age, and Maternal Level of Education of the Association Between Invasive Group B Streptococcus Disease and Epilepsy 0–22 Years After Birth eReferences [file jamanetwopen-e239507-s001.pdf]

Supplemental Online Content

Lykke MR, Sørensen HT, Lawn JE, Horváth-Puhó E. Long-term risk of epilepsy following invasive group B *Streptococcus* disease in neonates in Denmark. *JAMA Netw Open*. 2023;6(4):e239507. doi:10.1001/jamanetworkopen.2023.9507

eAppendix 1. Diagnostic Code Numbers of Group B *Streptococcus* and Anatomical Therapeutic Chemical Classification System Codes of Group B *Streptococcus* Illnesses, Epilepsy, and Anti-Epileptic Drugs

eTable 1. Overview of Exposure and Outcome-Codes

eTable 2. Overview of Antiepileptic Drugs Included in This Study, ATC Code and Names (Clobazam Not Included)

eAppendix 2. Data Sources

eAppendix 3. Directed Acyclic Graph (DAG)

eAppendix 4. Measures of Interaction on an Additive Scale

eAppendix 5. Screening and Treatment for Suspected GBS Infection During Pregnancy or After Birth

eTable 3. Incidence Rates of Epilepsy Among Children With iGBS and in the Comparison Cohort, With Epilepsy Defined Based on ICD-10 Codes Only

eTable 4. Effect Modification by Sex and Gestational Age of the Association Between Invasive Group B *Streptococcus* Disease and Epilepsy 0–5 Years After Birth

eTable 5. Effect Modification by Sex, Gestational Age, and Maternal Level of Education of the Association Between Invasive Group B *Streptococcus* Disease and Epilepsy 0–22 Years After Birth

eReferences

This supplemental material has been provided by authors to give readers additional information about their work.

© 2023 Lykke MR et al. *JAMA Network Open.*

eAppendix 1. Diagnostic code numbers of group B *Streptococcus* and Anatomical Therapeutic Chemical Classification System codes of group B *Streptococcus* illnesses, epilepsy, and anti-epileptic drugs.

eTable 1. Overview of exposure and outcome-codes.

Definition of iGBS disease in the Danish National Patient Registry	ICD-10	ATC codes
**iGBS**		
• Meningitis	G00.2 (streptococcal meningitis)<br>Patients with:<br>[P36.0 (sepsis of newborn due to GBS) or A40.1 (sepsis due to GBS)]<br>and<br>[G00.9 (bacterial meningitis, unspecified) or G03.9 (meningitis, unspecified)]	NA
• Sepsis	P36.0 (sepsis of newborn due to GBS)<br>A40.1 (sepsis due to GBS)	NA
**Epilepsy**	G40	N03A (antiepileptic drugs) or N05BA09 (clobazam)
• Focal	G 400-402	
• Generalized	G 403-404	
• Unspecified	G 405-409	

iGBS = invasive group B *Streptococcus*. ICD-10 = International Classification of Diseases, 10th revision. ATC = Anatomical Therapeutic Chemical Classification System. NA = non-applicable.

eTable 2. Overview of antiepileptic drugs included in this study, ATC code and names (Clobazam not included).

ATC code and sub-categories	Name
**N03AA Barbiturates and derivatives**	
N03AA01	Methylphenobarbital
N03AA02	Phenobarbital
N03AA03	Primidone
N03AA04	Barbexaclone
N03AA30	Metharbital
**N03AB Hydantoin derivatives**	
N03AB01	Ethotoin
N03AB02	Phenytoin
N03AB03	Amino(diphenylhydantoin) valeric acid
N03AB04	Mephenytoin

© 2023 Lykke MR et al. *JAMA Network Open.*

N03AB05	Fosphenytoin
N03AB52	Phenytoin, combinations
N03AB54	Mephenytoin, combinations
**N03AC Oxazolidine derivatives**	
N03AC01	Paramethadione
N03AC02	Trimethadione
N03AC03	Ethadione
**N03AD Succinimide derivatives**	
N03AD01	Ethosuximide
N03AD02	Phenosuximide
N03AD03	Mesusuximide
N03AD51	Ethosuximide, combinations
**N03AE Benzodiazepine derivatives**	
N03AE01	Clonazepam
**N03AF Carboxamide derivatives**	
N03AF01	Carbamazepine
N03AF02	Oxcarbamazepine
N03AF03	Rufinamide
N03AF04	Eslicarbazepine
**N03AG Fatty acid derivatives**	
N03AG01	Valproic acid
N03AG02	Valpromide
N03AG03	Aminobutyric acid
N03AG04	Vigabatrin
N03AG05	Progabide
N03AG06	Tiagabide
**N03AX Other antiepileptics**	
N03AX03	Sultiame
N03AX07	Phencemide
N03AX09	Lamotrigine
N03AX10	Felbamate
N03AX11	Topiramate

© 2023 Lykke MR et al. *JAMA Network Open.*

N03AX13	Pheneturide
N03AX14	Levetiracetam
N03AX15	Zonisamide
N03AX17	Stiripentol
N03AX18	Lacosamide
N03AX19	Carisbamate
N03AX21	Retigabine
N03AX22	Perampanel
N03AX23	Brivaracetam
N03AX24	Cannabidiol
N03AX25	Cenobamate
N03AX26	Fenfluramine
N03AX27	Ganaxolone
N03AX30	Beclamide

© 2023 Lykke MR et al. *JAMA Network Open.*

eAppendix 2. Data sources.

The Danish population is covered by a tax-funded health care system. Every resident has a unique 10-digit personal identification number, which is always included as a variable when recording health care services in the registries. Hence, information from various registries can be linked via the personal identification number.

**Data source**	**Variables**	**Use of data source**
The Civil Registration System[1]	Civil Personal Registration number	To generate the general comparison cohort<br>To identify family members<br>To define vital status, sex, date of birth, and immigration status
Statistics Denmark<br>- Education registries (including Ministry of Children and Education)[2]<br>- Income Statistics Register[3]<br>- Employment[4]<br>- Danish registers on personal labour market affiliation[5]	Marital status, income, highest achieved education, employment status, and social support<br>Grades from annual tests in primary school	To define socioeconomic position
The Danish National Patient Registry[6]	ICD codes and dates for diagnois of infections, epilepsy, febrile convulsions, and psychiatric disorders	To determine outcome, index dates, and the exposure cohort<br>To identify comorbidities
The Danish Medical Birth Registry[7,8]	Date of delivery, parity, birth weight, gestational age, multiplicity, delivery type	To determine variables for exposed and unexposed individuals
The Psychiatric Central Research Register[9]	ICD codes for psychiatric disease	To identify epilepsy
The Danish Registry of Causes of Death[10]	Data on causes of death	To determine cause-specific deaths
The Danish National Prescription Registry[11]	Data on prescription drugs	To identify epilepsy, depression, and other mental illnesses

Covariates and linked registries

Group	Covariates	Covariate type	Database
Child	Sex<br>Birthweight	Categorical<br>Categorical	The Civil Registration System[1], the Danish

© 2023 Lykke MR et al. *JAMA Network Open.*

	Calendar period of epilepsy diagnosis<br>Age at epilepsy diagnosis<br>Type of epilepsy	Categorical<br>Continuous<br>Categorical	Medical Birth Registry[7,8],<br>The Psychiatric Central Research Register[9] and the Danish National Prescription Registry[11]
Birth	Gestational age<br>Pregnancy type<br>Delivery type	Categorical<br>Categorical<br>Categorical	Danish Medical Birth Registry
Mother	Birth year<br>Age at birth<br>Age at epilepsy diagnosis<br>Education<br>Income	Categorical<br>Categorical<br>Continuous<br>Continuous<br>Continuous	Civil Registration System[1], Psychiatric Central Research Register[9] and the registries within Statistics Denmark.

All residents in Denmark have been assigned a unique personal identification number since 1968 allowing for individual level linkage across the Danish health and administrative registries. The CRS is an administrative register and was established in 1968. It contains individual-level information on all people residing in Denmark[1]. The Danish National Patient Registry (DNPR) contains records of all admissions to all Danish non-psychiatric hospitals since 1977, and outpatient clinic and emergency department visits since 1995[6]. The DNPR was used to identify the exposed cohort, outcome, and comorbidities by using the International Classification of Diseases, 10th revision (ICD-10) codes. The index date could therefore also be retrieved by using the DNPR. The Danish Medical Birth Registry (DMBR) was established in 1973 and includes data on all births in Denmark, including birth weight, gestational age, date of delivery, parity, and multiplicity[7]. The general population cohort was sampled from the DMBR and the CRS. Information regarding psychiatric diseases used for outcome assessment was obtained from The Psychiatric Central Research Register (PCRR). The PCRR holds information on, among others, psychiatric diagnosis, type of treatment, and type of referral, from 1970 onwards[9,12]. The Danish National Prescription Registry (NPR) contains data on prescription drugs and was used in this study to identify children as well as mothers with epilepsy[11]. ICD-10 codes for mental, behavioural, and nervous system disorders together with Anatomical Therapeutic Chemical Classification System (ATC) codes were used to identify patients diagnosed with epilepsy[6].

© 2023 Lykke MR et al. *JAMA Network Open.*

In Denmark, health care services are provided through a tax-funded health care system with coverage for all residents. Furthermore, all individuals receive social support if they are unemployed or unable to work. Depending on the life situation (i.e., age, living at home or by yourself, having a child, caring for a child on your own, mental disorders etc.) social services cover a yearly income between 90,000 and 246,000 DKK (~7500-32,800 Euro), and those people would fall into the poorest part of the population. All healthcare services would still be free of charge – also for their children.

In Denmark the tax-system makes it possible for the government to provide free education for all children in Denmark. After primary school the government provides each young individual continuing any education (any secondary education and/or higher education) with a monthly payment "state education support" (called SU), and the payment then depends on the level and the length of the education. All educations are free of charge if all requirements for the individual education are met. This means that regardless of your parent's income, you can get any (also tertiary) education in Denmark.

In terms of income, we have calculated the income quartiles yearly to handle inflation, i.e., separately for the mothers of children born in 1997, 1998, ….2017. The income is the yearly gross income from the year prior to childbirth and divided into 25th, 50th, and 75th percentile values. As an example, for a mother giving birth in 2017 the 25th percentile would correspond to ~180.000 DKK (~24.000 Euro), median ~290.000 DKK (~39.000 Euro) and 75th per-centile ~380.000 DKK (~51.000 Euro).

eAppendix 3 Directed acyclic graph (DAG).

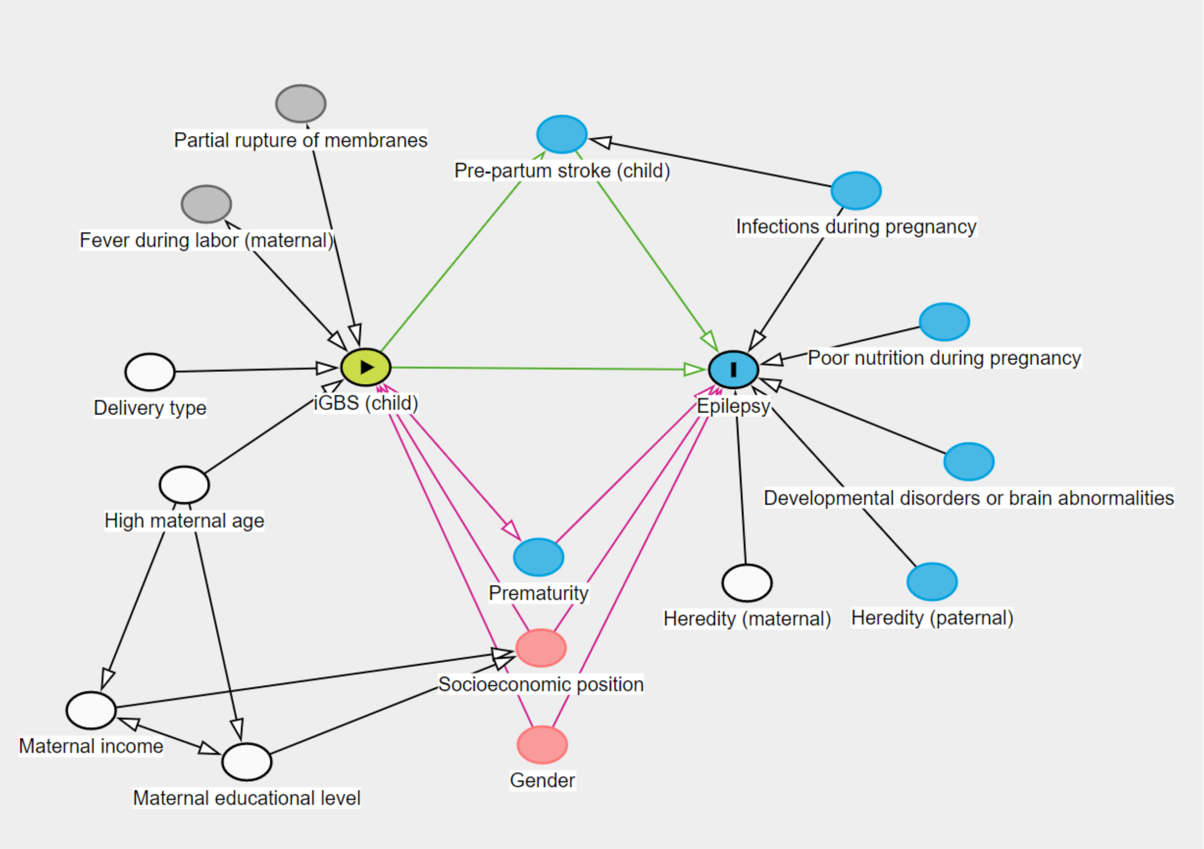

Based on the above DAG our covariates included: maternal age, maternal income level, maternal level of education, and maternal epilepsy status (heredity)(white circles). We adjusted for sex, prematurity, year of birth, and income. Pre-partum stroke in the child may act as an intermediate between iGBS and epilepsy. Thus, we did not adjust for this covariate in the main model as it would lower the true total effect. Causal pathways (direct and indirect) are plotted with green arrows and confounding pathways were plotted with red arrows. Factors associated with only iGBS or epilepsy are plotted with grey or included as covariates (white circles). Maternal infection during pregnancy, poor maternal nutrition during pregnancy, developmental disorders or brain abnormalities in the child or paternal epilepsy was predecessors of epilepsy, which we were not able to obtain sufficient data on. For prematurity, we cannot determine from our data whether a premature child was born premature due to iGBS or if iGBS induced premature birth. Therefore, we included prematurity in our effect modification analysis as well as in other statistical analyses.

The figure was created using daggity.net.

eAppendix 4: Measures of interaction on an additive scale.

For two dichotomous factors A and B, the following applies:

IR_{A+B+} is the incidence rate of disease if both risk factors, A and B, are present, whereas IR_{A-B-} is the incidence rate in the absence of both risk factors. (IR_{A-B+} - IR_{A-B-}) equals the "modifying variable" in our example.

Interaction contrast (IC):

$IC = IR_{A+B+} - IR_{A+B-} - IR_{A-B+} + IR_{A-B-}$

IC > 0 means positive interaction or more than additivity.

IC < 0 means negative interaction or less than additivity.

The attributable proportion (AP) of the combined effect that is due to interaction:

$AP = IC/IR_{A+B+}$

AP > 0 means positive interaction or more than additivity.

AP < 0 means negative interaction or less than additivity.

© 2023 Lykke MR et al. *JAMA Network Open.*

eAppendix 5. Screening and treatment for suspected GBS infection during pregnancy or after birth.

In Denmark, antibiotic prophylactic treatment during pregnancy against GBS is initiated if:

1) a pregnant woman experienced a serious clinical infection in a previous pregnancy.
2) she had a previous abortion in the second trimester or a stillbirth where GBS was suspected.
3) a previous preterm birth with suspected association to GBS.
4) or if birth has started before 35 full weeks of gestation.

Women are eligible for a PCR test for GBS if they have:

a) a birth at 35-36 full weeks of gestation.
b) a urine sample containing GBS at any time during pregnancy.
c) a preterm partial rupture of membranes for more than 18 hours, or fever above 38 C measured two times within a 30-minute interval or fever above 39 C measured once during delivery.

The following guidelines apply to the new-born child:

If the mother is eligible for any of the above mentioned points, the child must be observed in hospital for 48 hours. If the mother had fever during birth, the child will also have blood samples taken, and their c-reactive protein evaluated every hour. If the c-reactive protein is 35-50 mg/l a doctor will examine the child and eventually initiate antibiotic treatment. If c-reactive protein is above 50 mg/l antibiotics will be initiated.

© 2023 Lykke MR et al. *JAMA Network Open.*

eTable 3: Incidence rates of epilepsy among children with iGBS and in the comparison cohort, with epilepsy defined based on ICD-10 codes only.

Overall outcome	Comparison cohort	iGBS cohort	Adjusted HR (95% CI)
**Follow-up period**	**IR per 1000 person-years (95% CI)**	**IR per 1000 person-years (95% CI)**	
0–22 years	0.97 (0.82–1.11)	2.09 (1.41–2.78)	2.15 (1.50–3.08)
0–5 years	1.27 (1.00–1.54)	4.04 (2.49–5.60)	3.15 (2.03–4.89)
>5–22 years	0.79 (0.62–0.95)	0.93 (0.35–1.50)	1.17 (0.61–2.26)

Adjusted hazard ratio (HR) refers to HR adjusted for sex, prematurity, year of birth, and maternal income with 95% confidence interval (CI). IR = IR = incidence rate per 1000 person-years with 95% confidence interval (CI). iGBS = invasive group B *Streptococcus*.

© 2023 Lykke MR et al. *JAMA Network Open.*

eTable 4: Effect modification by sex and gestational age of the association between invasive group B *Streptococcus* disease and epilepsy 0–5 years after birth. Measure of effect modification on an additive scale (95% CI); attributable proportion (AP, %).

	**Comparison cohort**		**iGBS cohort**		**Effect modification on an additive scale *(Interaction contrast [95%CI]); AP [%]***	**HR (95% CI) for iGBS within strata of sex/prematurity**
	**Children with/without epilepsy, N**	**IR (95% CI)**	**Children with/without epilepsy, N**	**IR (95% CI)**		
**Girl**	45/6297	1.54 (1.09–1.99)	12/628	4.18 (1.81–6.54)	0.85 (-2.48–4.19); 15.9%	2.68 (1.42–5.06)
**Boy**	47/7822	1.30 (0.93–1.67)	17/775	4.79 (2.51–7.07)		3.70 (2.12–6.45)
**Premature (20–36 weeks)**	25/2926	1.94 (1.18–2.70)	10/296	7.61 (2.89–12–32)	3.22 (-1.85–8.29); 42.4%	3.75 (1.80–7.83)
**Term (≥ 37 weeks)**	67/11193	1.28 (0.97–1.58)	19/1107	3.72 (2.05–5.39)		2.92 (1.75–4.86)

HR was adjusted for sex, prematurity, year of birth, and maternal income.

eTable 5: Effect modification by sex, gestational age, and maternal level of education of the association between invasive group B *Streptococcus* disease and epilepsy 0–22 years after birth.

**Effect-modifier**		**Not exposed to iGBS**<br><br>**IR (95% CI)**	**Exposed to iGBS**<br><br>**IR (95% CI)**	**Effect modification on an additive scale (95% CI)**	**Attributable proportion**
**Sex**<br>Boy vs girl	Girl	1.26 (1.01–1.51)	2.36 (1.27–3.45)	0.22 (-1.28–1.72)	10.6%
	Boy	1.09 (0.88–1.29)	2.41 (1.42–3.39)		
**Gestational age**<br>Preterm vs term	Term	1.04 (0.87–1.21)	1.97 (1.23–2.71)	1.44 (-0.85–3.73)	37.1%
	Preterm	1.67 (1.24–2.10)	4.04 (1.92–6.15)		
**Maternal education**<br>Medium vs low	Low	1.29 (1.00–1.58)	2.04 (0.89–3.19)	0.30 (-1.26–1.87)	15.9%
	Medium	1.16 (0.93–1.38)	2.21 (1.21–3.20)		
**Maternal education**<br>High vs low	Low	1.29 (1.00–1.58)	2.04 (0.89–3.19)	1.38 (-1.51–4.26)	47.7%
	High	0.84 (0.41–1.27)	2.04 (0.89–3.19)		

© 2023 Lykke MR et al. *JAMA Network Open.*

eReferences

1. Schmidt M, Pedersen L, Sørensen HT. The Danish Civil Registration System as a tool in epidemiology. *Eur J Epidemiol* 2014; **29**(8): 541-9.
2. Jensen VM, Rasmussen AW. Danish Education Registers. *Scand J Public Health* 2011; **39**(7 Suppl): 91-4.
3. Baadsgaard M, Quitzau J. Danish registers on personal income and transfer payments. *Scand J Public Health* 2011; **39**(7 Suppl): 103-5.
4. The National Centre for Register-based Research. The employment classification module. Available from: http://econ.au.dk/the-national-centre-for-register-based-research/danish-registers/the-employment-classification-module-akm/. .
5. Petersson F, Baadsgaard M, Thygesen LC. Danish registers on personal labour market affiliation. *Scand J Public Health* 2011; **39**(7 Suppl): 95-8.
6. Schmidt M, Schmidt SA, Sandegaard JL, Ehrenstein V, Pedersen L, Sørensen HT. The Danish National Patient Registry: a review of content, data quality, and research potential. *Clin Epidemiol* 2015; **7**: 449-90.
7. Bliddal M, Broe A, Pottegård A, Olsen J, Langhoff-Roos J. The Danish Medical Birth Register. *Eur J Epidemiol* 2018; **33**(1): 27-36.
8. Knudsen LB, Olsen J. The Danish Medical Birth Registry. *Dan Med Bull* 1998; **45**(3): 320-3.
9. Munk-Jørgensen P, Mortensen PB. The Danish Psychiatric Central Register. *Dan Med Bull* 1997; **44**(1): 82-4.
10. Gjersøe P, Andersen SE, Mølbak AG, Wulff HR, Thomsen OO. [Reliability of death certificates. The reproducibility of the recorded causes of death in patients admitted to departments of internal medicine]. *Ugeskr Laeger* 1998; **160**(35): 5030-4.
11. Pottegård A, Schmidt SAJ, Wallach-Kildemoes H, Sørensen HT, Hallas J, Schmidt M. Data Resource Profile: The Danish National Prescription Registry. *Int J Epidemiol* 2017; **46**(3): 798-f.
12. Mors O, Perto GP, Mortensen PB. The Danish Psychiatric Central Research Register. *Scand J Public Health* 2011; **39**(7 Suppl): 54-7.

© 2023 Lykke MR et al. *JAMA Network Open.*
