# Supplementary material for: Long-term Risk of Epilepsy Following Invasive Group B Streptococcus Disease in Neonates in Denmark
Source: JAMA Netw Open. 2023 Apr 21;6(4):e239507. doi: 10.1001/jamanetworkopen.2023.9507 (PMC10122176; doi:10.1001/jamanetworkopen.2023.9507)
Supplement: Supplement 2. — Data Sharing Statement [file jamanetwopen-e239507-s002.pdf]

Data Sharing Statement

Lykke. Long-term Risk of Epilepsy Following Invasive Group B Streptococcus Disease in Neonates in Denmark. *JAMA Netw Open*. Published April 21, 2023. doi:10.1001/jamanetworkopen.2023.9507

Data
Data available: No

Additional Information
Explanation for why data not available: Data are only available through The Danish Health Data Authority
